# Supplementary material for: Systematic evidence and gap map of research linking food security and nutrition to mental health
Source: Nat Commun. 2022 Aug 8;13:4608. doi: 10.1038/s41467-022-32116-3 (PMC9359994; doi:10.1038/s41467-022-32116-3)
Supplement: Supplementary file 1 — Supplementary Information [file 41467_2022_32116_MOESM1_ESM.pdf]

SUPPLEMENTARY MATERIAL

Supplementary methods 1: Example search strategy from Medline. Search for CAB Global Health and PsychInfo databases are similarly constructed.

1. Ovid MEDLINE(R) and In-Process & Other Non-Indexed Citations and Daily <1946 to July 24, 2020> Searched 28th July 2020

1 ("mental health" or ((distress or stress) adj2 (psychological or psychosocial or mental)) or depression or wellbeing or "well being" or well-being or ((depressive or anxiety or anxious or affective or mood or mental) adj2 disorder*)).ti,ab,kw. (597107)

2 *depression/ or *depression, postpartum/ or *adaptation, psychological/ or *emotional adjustment/ or *survivorship/ or *mental health/ or *resilience, psychological/ or *mental disorders/ or *anxiety disorders/ or *mood disorders/ or *depressive disorder/ or *adjustment disorders/ or *stress disorders, traumatic/ or *affective symptoms/dh, ec, ep, eh, et, mo, pp, pc, rh, th or *psychological trauma/ or *stress, psychological/ (420912)

3 or/1-2 (811209)

4 *nutrition disorders/ or *child nutrition disorders/ or *infant nutrition disorders/ or *exp malnutrition/ or *fetal nutrition disorders/ or *severe acute malnutrition/ or *kwashiorkor/ or *wasting syndrome/ or *nutritional status/ (43295)

5 (nutrition* or nutrient* or micronutrient* or malnutrition or malnourish* or kwashiorkor or marasmus or undernutrition or ((iron or iodine or vitamin* or zinc) adj3 deficien*)).ti,ab,kw. (480349)

6 or/4-5 (487613)

7 *dietary supplements/ or *breast feeding/ or *diet/ or *feeding behavior/ or *bottle feeding/ or *food preferences/ or *weaning/ or *food supply/ or *famine/ or *eating/ or *maternal nutritional physiological phenomena/ or *prenatal nutritional physiological phenomena/ or *nutritional requirements/ or *Infant Nutritional Physiological Phenomena/ or *Child Nutritional Physiological Phenomena/ or *infant food/ or *infant formula/ (221063)

8 (diet* or ((food or eating) adj3 (pattern* or intake or shortage* or securit* or insecurit* or supplement* or access* or depriv* or belief* or consumption or cost* or purchas* or buy* or afford* or supplies or supply or availab*)) or "breast feed*" or "breast fed" or breastfeed* or breastfed or "bottle feed*" or "bottle fed" or ((infant* or child*) adj2 (feed* or food* or nutrition* or formula* or wean*))).ti,ab,kw. (687330)

9 or/7-8 (763547)

10 *growth disorders/ or *fetal growth retardation/ or *fetal development/ or *fetal viability/ or *developmental disabilities/ or *pregnancy outcome/ or *pregnancy complications/px or *premature birth/ or *birth weight/ or *fetal weight/ or *thinness/ or *failure to thrive/ or *exp infant, low birth weight/ or *exp infant, premature/ or *"child of impaired parents"/ or *child development/ (112556)

11 ((growth adj2 (disorder* or retard*)) or ((pregnan* or birth) adj3 outcome*) or birthweight or "birth weight" or ((premature or preterm or "pre term") adj2 (infant* or birth*)) or "failure to thrive").ti,ab,kw. (175595)

12 or/10-11 (247643)

13 ((obes* or overweight or "over weight" or underweight or "under weight" or ((body or bodily) adj2 (thin or thinness))) adj5 (nutrition* or nutrient* or micronutrient* or malnutrition or malnourish* or kwashiorkor or marasmus or undernutrition or ((iron or iodine or vitamin* or zinc) adj3 deficien*))).ti,ab,kw. (6950)

14 (*obesity/dh, ep, eh, et, pc, px, rh or *obesity, maternal/dh, ep, eh, et, pc, px, rh or *obesity, morbid/dh, ep, eh, et, pc, px, rh or *pediatric obesity/dh, ep, eh, et, pc, px, rh or *overweight/dh, ep, eh, et, pc, px, rh or *thinness/dh, ep, eh, et, pc, px, rh) and (nutrition/ or nutrition disorders/ or child nutrition disorders/ or infant nutrition disorders/ or exp malnutrition/ or fetal nutrition disorders/ or severe acute malnutrition/ or kwashiorkor/ or wasting syndrome/ or nutritional status/) (2177)

15 or/13-14 (8585)

16 *anthropometry/ or *apgar score/ or *"body weights and measures"/ or *body fat distribution/ or *body mass index/ or *body weight/ (60094)

17 (anthropometr* or "apgar scor*" or "body measur*" or "body mass index" or BMI).ti,ab,kw. (284745)

18 or/16-17 (314324)

19 6 or 9 or 12 or 15 or 18 (1538436)

20 ((systematic* or synthes*) adj3 (research or evaluation* or finding* or thematic* or report or descriptive or explanatory or narrative or meta* or review* or data or literature or studies or evidence or map or quantitative or study or studies or paper or impact or impacts or effect* or compar*)).ti,ab,kw. (347018)

21 ("meta regression" or "meta synth*" or "meta-synth*" or "meta analy*" or "metaanaly*" or "meta-analy*" or "metanaly*" or "metaregression" or "metaregression" or "logistic regression" or "methodologic* overview" or "pool* analys*" or "pool* data" or "quantitative* overview" or "research integration").ti,ab,kw. (452771)

22 (review adj3 (effectiveness or effects or systemat* or synth* or integrat* or map* or methodologic* or quantitative or evidence or literature)).ti,ab,kw. (425041)

23 ("meta ethnograph*" or "meta synthesis" or (synthesis and ("qualitative literature" or "qualitative research")) or "critical interpretive synthesis" or ("systematic review" and ("qualitative research" or "qualitative literature" or "qualitative stud*")) or "thematic synthesis" or "framework synthesis" or "realist review" or "realist synthesis" or "qualitative systematic review*" or "qualitative evidence synthes*" or (("quality assessment" or "critical appraisal" or "literature search*") and ("qualitative research" or "qualitative literature" or "qualitative stud*")) or (Noblit and Hare) or "meta narrative*" or "narrative synthesis").ti,ab,kw. (8246)

24 (random$ or placebo$ or single blind$ or double blind$ or triple blind$ or cohort$ or (case* adj2 control*) or ((cohort or follow up or follow-up) adj2 (control$ or series or report$ or study or studies))

or retrospective$ or ((quasi-experiment* or observ$) adj3 (study or studies or design))).ti,ab,kw. (2595318)

25 meta-analysis/ or "systematic review"/ or controlled clinical trial/ or randomized controlled trial/ or random allocation/ or equivalence trial/ or pragmatic clinical trial/ or case-control studies/ or retrospective studies/ or exp cohort studies/ or controlled before-after studies/ or cross-sectional studies/ or interrupted time series analysis/ or multivariate analysis/ or qualitative research/ or risk assessment/ (3427967)

26 or/20-25 (4958552)

27 3 and 19 and 26 (27169)

28 limit 27 to yr="2000 -Current" (24964)

Supplementary methods 2: Screening and coding guidance

Supplementary methods 2A: Screening inclusion and exclusion

Factors to include	Factors to exclude
**Literature Type**	
• Published scientific studies in English	• Conference abstracts<br>• Commentary and opinion pieces<br>• Grey literature reports<br>• Theses<br>• Studies reported in non-English languages
**Study Design**	
• Systematic reviews and meta-analyses<br>• Randomised control trials and quasi-experimental studies, or non-randomised experiments<br>• Cohort, case-control, or cross-sectional studies<br>• Secondary data analysis of survey data or surveillance data<br>• Mixed method studies<br>• Qualitative studies	• Non-systematic reviews<br>• Non-analytical studies (prevalence studies that only describe the presence of mental health or nutrition indicators without attempting to find an association between the two factors)<br>• Hypothetical modelling studies<br>• Medical case reports (less than 10 subjects) or case studies that are not qualitative studies
**Population**	
• General populations in any geographic location<br>o These may include specific age groups, specific genders or specific life-course stages such as pregnancy or older people<br>o Populations defined by certain equity aspects, such as low SES, income, occupations, or places of residence.<br>o Special populations including refugee status and prisoners will be included with a separate code	• Human populations<br>• Populations defined by specific health conditions such as heart disease, hypertension, diabetes, dementia, coeliac, anorexia, preterm birth, HIV, depression diagnosis, etc.<br>• Populations being treated in a clinical therapeutic setting, hospital settings<br>• Extremely niche populations with specific nutritional requirements such as professional athletes.
**Food and Nutrition Security Factors**	
**Food and Diets:**<br>Any measure of the following factors:<br>• Food security including food availability, food supply, food affordability, access to food and food utilization.<br>• Food intake patterns, including breakfast consumption and Mediterranean diet for example<br>• Dietary intake of food groups, specific nutrients or general supplement use<br>• Nutrient status including biomarkers and diagnosed deficiency diseases<br><br>**Birth outcomes that relate to growth**	• Alcohol intake is not considered a FNS measure<br>• Stimulants such as caffeine, nicotine, and other stimulant foods or drugs are not considered an FNS measure<br>• Single food items not included (e.g. chocolate, broccoli, walnuts, herbal remedies)<br>• Proprietary and patented supplemental formulas or products<br>• Probiotics and microbiome related formulas or microbiome status indicators

• Intrauterine growth restriction and small-for-gestational age<br>• Birth weight<br>• Birth length<br>• Head circumference<br><br>**Infant and Young Child Feeding**<br>• Early initiation of breastfeeding<br>• Exclusive breastfeeding under 6 months<br>• Continued breastfeeding at 1 year<br>• Introduction of solid, semi-solid or soft foods<br>• Minimum dietary diversity<br>• Minimum meal frequency<br>• Minimum acceptable diet<br>• Consumption of iron-rich or iron-fortified foods<br>• Children ever breastfed<br>• Continued breastfeeding at 2 years<br>• Age-appropriate breastfeeding<br>• Predominant breastfeeding under 6 months<br>• Duration of breastfeeding<br>• Bottle feeding<br>• Milk feeding frequency for non-breastfed children<br><br>**Anthropometry**<br>• Relative height, relative weight<br>• Wasting, weight-for-height<br>• Stunting, height-for-age, length-for-age<br>• Underweight, weight-for-age, Mid-Upper Arm Circumference<br>• Obese, overweight (BMI or body mass index)<br>• Underweight<br>• Waist circumference<br>• Body composition such as scans and visceral adiposity measures	• Anorexia nervosa or bulimia is not considered as an FNS measure of food intake or eating patterns<br>• Emotional eating, disordered eating, night-time eating, binge eating, fasting, dieting<br>• Studies that only describe understanding of diets and nutrition rather than actual intake<br>• Perceptions, preferences and attitudes about food, weight, etc.<br>• Hormones, hormonal therapy<br>• Weight loss interventions, low calorie diets, weight loss diets<br><br>• Premature delivery<br>• Apgar scores<br>• Neurological, emotional and social developmental measures<br>• Measures that relate to the development of specific physiological functions e.g. immune function<br><br>• Studies that only describe understanding of IYCF practices rather than actual practices or feelings about breastfeeding and psychological elements, yes/no breastfeeding questions outside of standard indices<br><br>• Change in weight (gestational weight gain) or weight trajectories
**Mental Health**	
• Any experiential measure of mental wellbeing including Health-Related Quality of Life, mental quality of life, perceived mental wellbeing<br>• Any measures of depression, such as:<br>o Beck's Depression Inventory<br>o CES-D<br>o EPDS<br>o GHQ-5, 12<br>o Goldberg - 15<br>o HADS-A<br>o HADS-D<br>o Kessler 6, 10<br>o Kitgum<br>o MINI	• Studies that only refer to specific psychoses or disorders such as bipolar, Alzheimer's, Parkinson's, schizophrenia and addiction/smoking disorders<br>• Studies in which the exposure is anti-depressants or other medication rather than the condition itself<br>• Stressful event indices without accompanying experiential measures<br>• Post-traumatic stress or specific occupational stress (based on type of work, etc.) is not be included on its own<br>• Internalizing/externalizing behaviours on their own

- Negative/positive experiences scale
- PDSS
- PHQ-9
- SC-90-R
- SRQ-20
- SSQ
- ZSDS

- Perceived measures of stress
- Any measures of anxiety
- Affective disorder or affective symptoms
- Mood disorders

- Cortisol as an isolated measure of stress
- Happiness, general satisfaction (without specific mental health measures), or composite tools where the mental health components cannot be extracted.

Supplementary methods 2B: Flowchart for screening studies

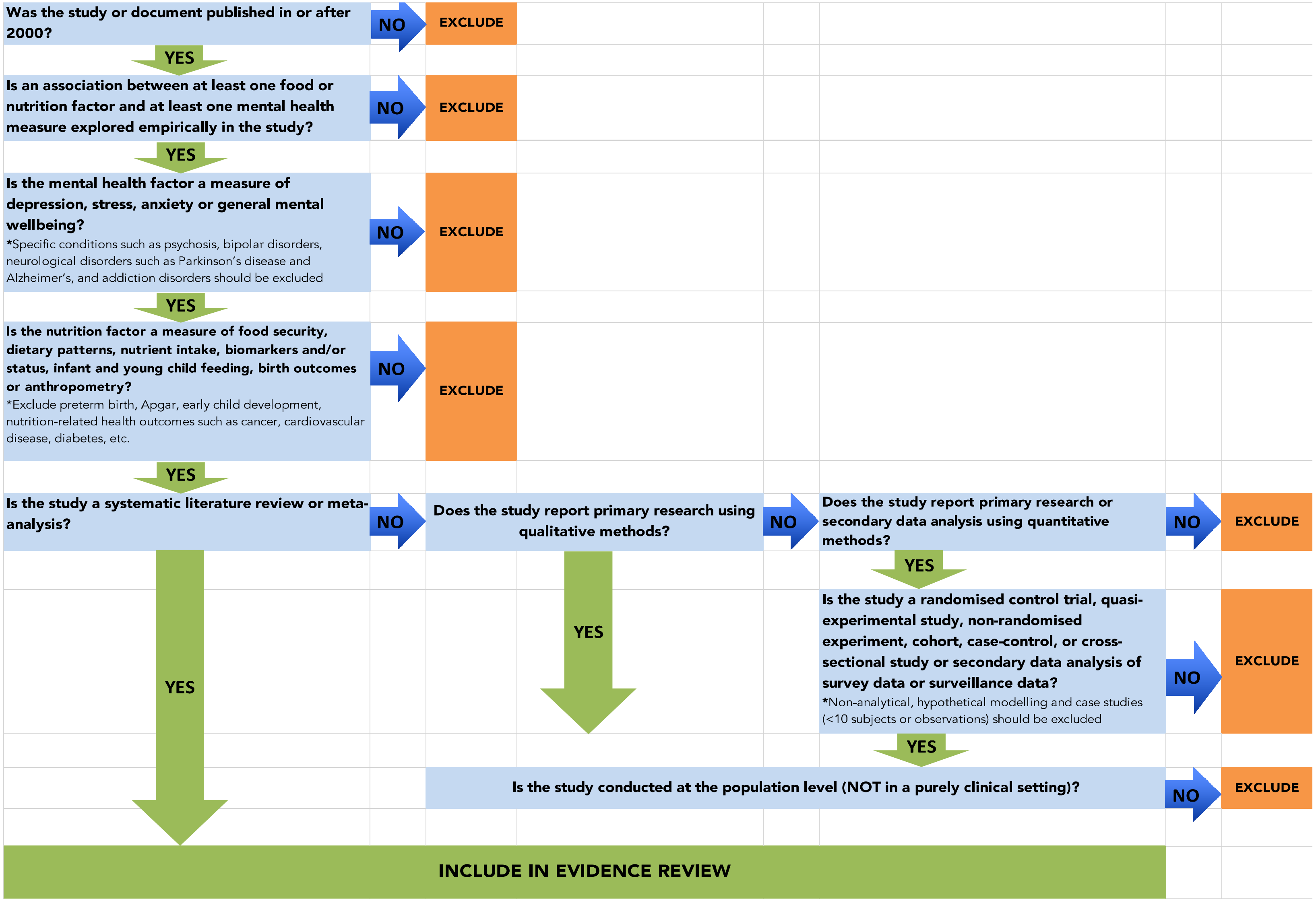

Supplementary methods 2C: Coding form structure

DROP DOWN HEADINGS	TICK BOX SECTION/FREE TEXT BOXES	TICK BOX SECTION/FREE TEXT BOXES
**PHASE I**		
**STUDY PUBLICATION DATE**		
	Will provide a full drop-down menu of years from 2000-present	
**STUDY LOCATION (REGION)**		
	Will provide a full drop-down list of regions, as well as other geographic and political categories such as global (more than 10 involved countries), Low- and Middle-Income Countries, Organization for Economic Cooperation and Development countries, or Africa.	
**STUDY LOCATION (COUNTRY)**		
	Will provide a full drop-down list of countries with checkbox, plus code for Developing countries, Europe, Africa and Global where more than 5 regional studies	
**STUDY DESIGN**		
	Systematic review<br>Meta-analysis<br>Experimental<br>Quasi-experimental<br>Longitudinal (cohort, panel, surveillance)<br>Cross-sectional study<br>Case-control study<br>Mixed methods<br>Qualitative<br>Ecological<br>Nested Design	
**EXPOSURE**		
	Mental Health<br>Food Security and Nutrition	
**OUTCOME**		
	Mental Health<br>Food Security and Nutrition	
**FOOD SECURITY AND NUTRITION MEASURE(S)**		
	Food security	**EXAMPLES, those defined *a priori* (not exhaustive):**<br>Food Consumption Score (FCS)<br>Latin American Food Insecurity Scale (ELCSA)<br>Food Insecurity Experiences Scale (FIES)<br>(HDDS)Household Food Insecurity Access Scale (HFIAS)<br>Household Hunger Score (HHS)<br>Months of Adequate Household Food Provisioning (MAHFP)<br>USDA Household Food Security Scale<br>Other (Write in info box)

Nutritional risk	Nutritional Risk Index<br>Mini Nutritional Assessment<br>SCREEN II: Seniors in the Community: Risk Evaluation for Eating and Nutrition
Diets - Dietary patterns including intakes of specific foods or food groups	Dietary Patterns (Healthy, unhealthy, Glycemia, etc.)<br>Mediterranean diet<br>Dietary Inflammatory Index<br>Household Dietary Diversity Score<br>Women's Dietary Diversity<br>Specific food groups
Dietary intakes of specific nutrients	Vitamins - Specify<br>Minerals - Specify<br>Macronutrients - Specify<br>Polyphenols and flavonoids (anthocyanins)<br>Other nutrients (Write in info box)
Nutrient biomarkers	Vitamins - Specify<br>Minerals - Specify<br>Macronutrients - Specify<br>Polyphenols and flavonoids (anthocyanins)<br>Other nutrients (Write in info box)
Infant and Young Child Feeding	Breastfeeding<br>Complementary feeding<br>Minimum Dietary Diversity<br>Minimum Meal Frequency<br>Minimally Adequate Diets<br>Other IYCF (Write in info box)
Birth Outcomes	Birth weight<br>Small-for-Gestational age (SGA/IUGR)<br>Birth length<br>Head circumference
Anthropometry	Body Mass Index (BMI)<br>Body Composition<br>Waist circumference<br>Relative height, stunting, HAZ<br>Relative weight, wasting, WAZ<br>Mid-Upper Arm Circumference (MUAC)<br>Underweight<br>Other anthropometry (Write in info box)
**FOOD SECURITY AND NUTRITION POPULATION**	
Children under 5	Newborns<br>Children 6-24 months<br>Children 24-60 months
Children 5-12	
Adolescents	
Women of Reproductive Age (WRA)	Pregnant women<br>Postnatal women<br>Perinatal Women<br>Mothers<br>Other WRA (prenatal, mixed, etc.)
Parents	
Household	

Male<br>Female<br>Elderly	
**MENTAL HEALTH MEASURE(S)**	
Depression	**EXAMPLES, those defined *a priori* (not exhaustive):**<br>BDI/Beck<br>CES-D<br>EPDS<br>Goldberg<br>Kessler<br>PDSS<br>PHQ-9<br>PDQ<br>PRIME-D<br>SC-90-R<br>SCID-I<br>Self report of diagnosis<br>Medical records<br>Clinical or diagnostic interview<br>Other depression measure (Write in info box)<br>Mixed depression measures (reviews)
Hybrid/Multiple domains	GHQ<br>HADS<br>HSCL<br>MINI<br>SCL-90-R<br>SDQ<br>SRQ-20<br>SSQ<br>Other hybrid measure (Write in info box)<br>Mixed hybrid measures (reviews)
Anxiety	GAD, GAD-7<br>HADS-A<br>PDSS<br>Self report of diagnosis<br>Medical records<br>Clinical or diagnostic interview<br>Other anxiety measure (Write in info box)<br>Mixed anxiety measures (reviews)
Stress	Kessler<br>PSS<br>Other stress measure (Write in info box)<br>Mixed stress measures (reviews)
Mental wellbeing	Negative and positive experiences scale<br>SF-36<br>SPANE<br>WHOQoL<br>Other QoL measure (Write in info box)<br>Mixed QoL measures (reviews)
**MENTAL HEALTH POPULATION**	

	Children under 5	
	Children 5-12	
	Adolescents	
	Women of Reproductive Age (WRA)	Pregnant women<br>Postnatal women<br>Perinatal Women<br>Mothers<br>Other WRA (Prenatal, mixed, etc.)
	Parents	
	Male	
	Female	
	Elderly	
**ANALYSIS ADJUSTMENT**		
	Adjusted analysis	Other variables are controlled or accounted for in the final results model
	Not adjusted analysis	No other variables are controlled or included in the final results model
	Pooled analysis	*For Reviews* - pooled narrative or quant summary
**SAMPLE SIZE**		
	1-10	EXCLUDE
	11-100	Specify exact number
	101-500	Specify exact number
	501-1,000	Specify exact number
	1001-5000	Specify exact number
	>5,000	Specify exact number
	Reviews (Studies included in review)	1-5, 6-10, 11-15, 16-20. 21-30, 31-40, 41-50, 51-100, >100

Supplementary methods 2D: Coding form guidance

CODING CATEGORY	INSTRUCTIONS
**Publication date**	Choose the year of publication, according to the bibliographical record. All included studies should be published between 2000 and 2020.
**Study location (region)**	Select the region of the study (Africa, South America, Arab nations, etc.). If the study is from a nation classed as a Low- or Middle-Income Country (LMIC) or 'developing' based on the classification listed in the description of the code, please select 'LMIC'. If the country is part of the OECD nations listed in the code description, select 'OECD'. If the study spans more than 5 countries in two or more regions, select 'Global'.<br><br>For reviews, select the regions/groups based on inclusion criteria. For example, if a review says they searched for studies only in LMICs, but then included only studies from Asia and Africa, do NOT select on included study regions, rather simply select 'LMIC'.
**Study location (country)**	Choose the country or countries where the studies are carried out. Select all that apply.<br><br>For reviews, select the countries based on inclusion criteria. For example, if a review says they searched for studies only in LMICs, but then included only studies from Brazil and Malawi, do NOT select on included study countries, rather simply select 'Global' in regions.
**Study design**	Systematic reviews and meta-analyses are listed separately. Please select one or both that apply.<br><br>If not a review, select *one* of the study designs that most closely matches the description in the methods. If the study presents several analyses, for instance in a cohort where some analysis is cross-sectional and some is longitudinal, choose the 'highest order' design – 'Longitudinal' in this case. If unclear, or an approach not listed, select 'other study design'.<br><br>The last option is 'Nested design'. This code should be used if the primary analysis presented is a certain study type, but is taken from or nested within a parent study. This would include nested case-control studies (a case control nested within a cohort), a trial within a cohort (TWIC) or a Study within a Trial (SWAT). Select this code if the study is nested within another design, but code on the primary analysis. For instance, if the analysis presented is cross-sectional, but comes from a trial, select "Nested design".
**Exposure**	Choose either 'mental health' or 'food and nutrition security' as the **hypothesized** exposure, explanatory, risk or independent ('x') variable. Use the study aims and objectives, the methods, and the results and/or discussion to ascertain the hypothesis, regardless of whether authors prove causality or directionality using data.<br><br>If a bidirectional association is examined, or the exposure-outcome hypothesis is reversed and then tested in both ways, select both "Food and nutrition security' AND 'Mental health' as the exposure.
**Outcome**	Choose either 'mental health' or 'food and nutrition security' as the **hypothesized** outcome or dependent, response, or y- variable. Use the study aims and objectives, the methods, and the results and/or discussion to ascertain the hypothesis, regardless of whether authors prove causality or directionality using data.<br><br>If a bidirectional association is examined, or the exposure-outcome hypothesis is reversed and then tested in both ways, select both "Food and nutrition security' AND 'Mental health' as the outcome.
**Food and Nutrition Security (FNS) measurement**	This section is to indicate the food or nutrition security aspect measured or described. Choose all that apply. It is sectioned into:<br>- Food scarcity (food insecurity and nutritional risk)<br>- Diets, dietary patterns and food groups<br>- Specific nutrients measured through intake<br>- Specific nutrients measured by biomarker assessment

	- Infant and Young Child Feeding (IYCF) measures defined by the WHO<br>- Birth outcomes, including birthweight (BW) and Gestational Age (SGA or IUGR), but not including preterm birth.<br>- Anthropometry (measures of the body indicative of nutrition), whether child or adult<br>Within these sections, select from the specific tools and measures listed. If there is a measure that is unclear or unlisted, use the 'other' categories. Please copy and paste the name of the tool or measure into the info box for all 'other' selections. We will add to the specified list if there is more than one report using the tool or measure.<br><br>If BMI or other FSN factors are included as confounders but not within the main hypothesis, Do NOT code these. Only code FNS measures that are described in the main associations of interest or hypothesis. If it is an exploratory analysis looking at all 'predictors' of MH, these can all be included.
**FNS population**	Choose the population group of the FNS measure. If there is crossover between categories, select all that apply. For example, if the children included are measured from birth to 5 years, choose both 'newborns' and 'children under 5'.<br><br>If there are multiple measures or multiple times of assessments that cross the population categories, select all time points at which the independent variable is compared with the dependent. For instance, if a cohort of men are followed from adolescence to old age, and they assess the relationship of Vitamin D with mental health at age 30, 50, and 75, then select 'Adult', 'Male' and '65+'. If they also assessed and analysed both at age 16, then select 'Adolescents' as well.<br><br>The 'male' and 'female' categories can be used if there is only one sex included in the sample. For example, in a study of elderly women, you would select both 'elderly' and 'female'. If both sexes are included, do not select 'male' or 'female' – leave these blank.<br><br>For Pregnant Women and Mothers (PWM), select the appropriate category. Within PWM, there is an 'Other PWM' group. This would apply in a study of mothers who were assessed for diets or nutrient biomarkers 'prenatally' but not in pregnancy, per se. Do not code 'Female' as well as PWM, as it is implicit in PWM.<br><br>As we are looking at many indicators of both mental health and FSN, linked through models in which the hypothesized relationship could be presented variously, we will indicate the population of the FSN measure separately from the Mental health (MH) measure. For example, some studies examine the risk of poor MH later in life from stunting or food insecurity at birth, while others examine poor mental health in mothers and the FSN outcomes of their children.<br><br>**Code birth outcomes of adults in a study sample as the study sample population.** For example, if middle-aged women are followed as the participants, and their birth outcomes analysed by using their birth records, these FSN populations would still be 'Adult' and 'Female'. In other words, for a single population studied, the FSN populations and MH population should match. For studies of mothers/parents and offspring, then these measures would differ.
**Mental health (MH) measurement**	This section is to indicate the mental health aspect measured or described. Choose all that apply. It is sectioned into:<br>- Depression<br>- Hybrid measures or tools/multiple or mixed domains of mental health<br>- Anxiety<br>- Stress<br>- Mental wellbeing (and Quality of Life (QoL))<br><br>Within these sections, select from the specific tools and measures listed. If there is a measure that is unclear or unlisted, use the 'other' categories. Please copy and paste the name of the

	tool or measure into the info box for all 'other' selections. We will add to the specified list if there is more than one report using the tool or measure.<br><br>For reviews on 'depression' or 'anxiety' where any valid measure of these MH conditions are allowed, use the 'Mixed measures' codes at the bottom of the list.
**MH population**	Choose the population group of the MH measure. If there is crossover between categories, select all that apply. For example, if the study is in adults over 50, select both 'Adults' and '65+'. There are no categories for children under 5, as mental health states are not normally measured for infants and very small children.<br><br>If there are multiple measures or multiple times of assessments that cross the population categories, select all time points at which the independent variable is compared with the dependent. For instance, if a cohort of men are followed from adolescence to old age, and they assess the relationship of Vitamin D with mental health at age 30, 50, and 75, then select 'Adult', 'Male' and '65+'. If they also assessed and analysed both at age 16, then select 'Adolescents' as well.<br><br>The 'male' and 'female' categories can be used if there is only one sex included in the sample. For example, in a study of school-aged boys, you would select both 'children 5-12' and 'male'. If both sexes are included, do not select 'male' and 'female' – leave these blank.<br><br>For Pregnant Women and Mothers (PWM), select the appropriate category. Within PWM, there is an 'Other PWM' group. This would apply in a study of mothers who were assessed for mental health 'prenatally' but not in pregnancy, per se. Do not code 'Female' as well as PWM, as it is implicit in PWM.<br><br>As we are looking at diverse indicators of both MH and FSN, linked through models in which the hypothesized relationship could be presented variously, we will indicate the population of the FSN measure separately from the MH measure. For example, some studies examine diets as a result of anxiety and stress in a single cohort, while others examine household food insecurity and the impact on women, or children.
**Analysis**	Select whether the most final results presented are 'Not adjusted' or 'Adjusted'. If authors present, for example, Pearson chi-square correlations without stratification or other variables considered, this would be 'Not adjusted'. If they present a multivariable model with adjustment for age, cluster, health history, etc., this would be 'adjusted'. If they present a stratified model without additional confounders, this is NOT considered adjusted. They would need to include two or more explanatory variables together in a model to be considered adjusted (E.g. Depression is the outcome variable (y), and the exposure variables are BMI (x1), and age (x2), then $y = \beta 0 + \beta 1\ x1 + \beta 2\ x2$).<br><br>For reviews, there is a code for 'pooled analysis'. As long as the authors have presented summary (narrative or quantitative) results from included studies, select this box.
**Sample size**	Select the sample size of the study. If there are various analyses presented with different sample sizes, choose the most 'final' model, or the model from which the results are extracted. Write in the exact sample size in the 'Info' box.

Supplementary methods 3

Supplementary methods 3A: Categories, inclusion and exclusion examples of food security and nutrition measures

**Category**	**Subcategories**	**Included**	**Examples (illustrative, not comprehensive)**	**Excluded**	**Justification**
Food scarcity	Food insecurity and famine	Quantitative measure of food insecurity, exposure to famine or nutritional risk (usually in elderly) using scales, indicators, questionnaires, or qualitative investigation	Food Consumption Score (FCS)<br>Food Insecurity Experience Scale (FIES)<br>United States Department of Agriculture (USDA) module		
	Nutritional risk		Mini Nutritional Assessment (MNA)<br>Malnutrition Universal Screening Tool (MUST)<br>Nutritional Risk Index (NRI)	· Frailty indices without separate nutritional component	· Frailty alone is not necessarily nutrition-related
Diets	Food Groups	Quantitative measures of specific food groups or dietary patterns or quality using food frequency questionnaires, dietary recall, food journals, consumption scores, indices, principal components analysis (PCA) or qualitative investigation	Food groups such as fruits and vegetables, sweets, unprocessed foods, fish, dairy or eggs	· Single-item foods (e.g. avocadoes, seaweed, walnuts)<br>· Specialized or proprietary foods or formulas<br>· Alcohol, caffeine, or stimulant foods	· Single consumed food items are not likely to be indicative of nutrition overall or generally representative, conflicts of interest as often funded by food lobby<br>· Not generalizable, conflicts of interest<br>· Stimulants and stimulant foods will have independent effect or interaction with mental health
	Dietary patterns and quality		Dietary patterns (grouped using fixed indices, PCA, nutrient or food group clusters, etc.) such as healthy or unhealthy diets, dietary diversity, adherence to dietary recommendations, traditional diets, Dietary Inflammatory Index (DII)	· Perceptions, attitudes and behaviors about food or diets with no intake component (e.g. eating family dinners, weight loss, dieting)<br>· Emotional eating, disordered eating, night-time eating, binge eating, emotional eating scales	· Not direct measures of food security or nutrition<br>· Emotional, binge eating are by themselves markers of mental health rather than nutrition<br>· Not direct measures of food security or nutrition

				(e.g. Power of Food index)<br>· Studies that only describe understanding of diets and nutrition rather than actual practices.	
Nutrient intakes	Vitamins	Quantitative measures of specific nutrients (via food and/or supplements) ascertained using food frequency questionnaires, dietary recall, food journals, consumption scores, indices or qualitative investigation	Intake of Vitamin A, Vitamin D, or reviews of B vitamins		
	Minerals		Intake of Selenium, Zinc, or Calcium	· Toxins, non-nutritive minerals	· Not direct measures of food security or nutrition
	Macronutrients		Intake of total calories, sugar, polyunsaturated fatty acids (PUFA), fats (e.g. triglycerides)	· Amino acids, specific proteins	· Often confounded with metabolism versus general measures of food security and nutrition; too granular for scope of project
	Polyphenols, flavonoids, antioxidants		Anthocyanins, phytochemicals, foods rich with antioxidants		
	Supplements		Supplementation with Iron and folic acid, Vitamin D, or multivitamins	· Probiotics<br>· Patented, proprietary, performance or specialized formulas	· Not generalizable, conflicts of interest
	Mixed reviews, other				
Nutrient biomarkers	Vitamins	Nutrient levels measured via biological assessment in blood, urine, fat, or other metabolomic methods	Serum ferritin, omega-3 PUFA, retinol or folate		
	Minerals		Serum Magnesium, Iodine, or Sodium	· Hormones, hormonal therapy	· Not direct measures of food security or nutrition
	Macronutrients		High-density lipoproteins in fat tissue, sugars or fiber		
	Polyphenols, flavonoids, antioxidants		Flavonoids, total antioxidant capacity		
	Mixed reviews, other				
Infant and young child feeding [IYCF]	Breastfeeding	Quantitative or qualitative measure of initiation, duration or exclusivity of breastfeeding	Exclusive breastfeeding at six months, initiation of breastfeeding in the first 48 hours after birth, length of time breastfeeding	· Studies that only describe understanding of IYCF practices rather than actual practices, intentions to breastfeed or feelings about breastfeeding, breastfeeding questions outside of	· Not direct measures of food security or nutrition

				standard indices, such as breastfeeding difficulty ratings	
	Child diets and complementary feeding	Quantitative or qualitative measures of complementary feeding practices based on standard WHO indicators, including introduction of complementary foods, dietary adequacy, meal frequency and dietary diversity	Minimally acceptable diets for children, dietary diversity for children, food groups eaten by children	· Attitudes and preferences about child feeding, knowledge scores with no measured child feeding behaviors	· Not direct measures of food security or nutrition
	Mixed reviews, other				
Birth outcomes (nutrition-related)	Birth weight	Quantitatively measured nutrition-related birth outcome, or qualitative assessment of birth outcomes linked specifically to nutrition	Low birth weight, very low birth weight	· Preterm birth and premature delivery	· Risk factors for and outcomes of preterm birth are often unrelated to nutrition, including factors such as high blood pressure, diabetes, multiple gestations, history of preterm birth, alcohol use or ethnicity
	Birth length		Length at birth	· Apgar scores	· Not direct measures of food security or nutrition at birth (general checklist of newborn health)
	Small-for-gestational age (SGA), Intrauterine Growth Restriction (IUGR)		Full term infants weighing less than 2500 grams	· Neurological, emotional and social developmental measures	· Related to nutrition but outside the scope of project
	Head circumference		-2 standard deviations below the WHO reference values at birth	· Measures that relate to the development of specific physiological functions e.g. immune function	· Often confounded by other factors, not direct measures of food security or nutrition
	Mixed reviews, other				

Anthropometry	BMI	Quantitative physical measurement of the body using scales, tapes, or sensors, or any qualitative ascertainment of growth, overweight or obesity related to mental health	BMI according to Asian classification of overweight, obese according to >30 BMI	· Weight change, weight loss or weight trajectories	· Often confounded by other factors, not direct measures of food security or nutrition
	Body composition		Measures of adiposity, skin fold tests, visceral fat scans		
	Body Ratios		Waist-to-hip ratios (WHR), leg length, sitting height		
	Circumference (waist, hip, calf, thigh)		Waist or hip circumference		
	Mid-Upper-Arm Circumference (MUAC)		Severe Acute Malnutrition in children defined by <115mm MUAC		
	Relative height, stunting, Height-for-Age Z-score (HAZ)		Growth faltering, severe stunting (<-3 z-score)		
	Relative weight, wasting, Weight-for-Height Z-score (WHZ)		Moderate acute malnutrition (MAM) (<-2 z-score), chronic MAM		
	Underweight, Weight-for-Age Z-score (WAZ)		Adolescent underweight		
	Mixed reviews, other				

Supplementary methods 3B: Categories, inclusion and exclusion examples of mental health measures

**Category**	**Subcategories**	**Included**	**Examples (illustrative, not comprehensive)**	**Excluded**	**Justification**
Depression	Screening	Any measures of depression	- Center for Epidemiological Studies-Depression (CES-D)<br>- Edinburgh Postpartum Depression Scale (EPDS)<br>- Geriatric Depression Scale (GDS)	· Studies in which the exposure is anti-depressants or other medication rather than the condition itself	· Not a direct measure of mental health
	Self-report, prescription proxy for diagnosis, medical records				
	Clinical, diagnostic assessment				
Hybrid measures (depression+)	Screening	Studies examining two or more of the	- Child Behaviour Checklist<br>- General Health Questionnaire	· Studies that only refer to specific psychoses or	· Not generalisable to other populations

	Self-report, prescription proxy for diagnosis, medical records	mental health measures above and/or investigate mood disorders or other affective symptoms	(GHQ)<br>- Hospital Anxiety and Depression Scale (HADS)<br>- Strengths and Difficulties Questionnaire (SDQ)<br>- Clinical/diagnostic interview	disorders such as bipolar, dementia, Alzheimer's, Parkinson's, schizophrenia and addiction/smoking disorders	
	Clinical or diagnostic assessment			· Eating disorders	· Confounded with food intake
	Qualitative				
Anxiety	Screening	Any measures of anxiety	- State-Trait Anxiety Inventory (STAI)<br>- Self report of diagnosis<br>- Clinical/diagnostic interview		
	Self-report, prescription proxy for diagnosis, medical records				
	Clinical, diagnostic assessment				
Stress	Screening	Any measures of perceived stress	- Kessler stress inventory<br>- Life event inventories<br>- Perceived Stress Scale (PSS)<br>- Single question screening	· Stressful event indices without accompanying experiential measures	
	Clinical, diagnostic assessment			· Post-traumatic stress or specific occupational stress (based on type of work, etc.) is not be included on its own	· Not generalisable to other populations
	Qualitative			· Cortisol as an isolated measure of stress	· Cortisol fluctuates for reasons not related to experienced stress (early in the morning, during birth, in some forms of exercise)
Mental wellbeing	Screening	Any experiential measure of mental wellbeing, including health-related quality of life, mental quality of life, perceived wellbeing	- EuroQol 5 dimension questionnaire (EQ-5D)<br>- Short Form 36 Health Survey Questionnaire (SF-36, Rand-36)<br>- Short Form 12 Health Survey Questionnaire (SF-12)	· Happiness, general satisfaction (without specific mental health measures), or composite tools where the mental health components cannot be extracted.	· Not a direct measure of mental health
	Qualitative			· Internalizing/externalizing behaviors in adults or without other measures	

**Screening**	Indicators of mental health illness made up of a set of symptom-based items or questions, usually with a classification system based on probability against diagnosis
**Self-report, prescription proxy for diagnosis, medical records**	- Questionnaires including items where respondents answer whether they have received a diagnosis from a medical professional<br>- Prescriptions for medications specifically for mental health problems from a medical provider as a proxy for diagnosis<br>- Medical records indicating mental health diagnosis
**Clinical or diagnostic assessment**	Clinical or diagnostic assessment either via trained medical professional or validated computer-assisted interview conducted within the study population
**Qualitative**	Qualitative investigation based on thematic analysis of group or individual interviews
**Mixed measures for reviews**	A group of studies where the ascertainment method of the mental health construct varies between studies, for instance in a meta-analysis.

Supplementary results 1

**MEASURES OF FOOD SECURITY & NUTRITION**	
**Food security**	163
Cornell-Radimer hunger scale	3
ELCSA: Latin American Food Insecurity Scale	2
FCS: Food consumption Score	2
Famine, hunger	9
FIES: Food Insecurity Experience Scale	5
HFIAS: Household Food Insecurity Access Scale	11
LMIC scale (Swindale, Frongillo)	2
USDA HHFS: Household Food Security Scale	70
1 or 2 item screen	32
Qualitative	5
Mixed Food Security measures (reviews)	9
Other Food Security measure	16
**Nutritional risk, famine**	70
Determine Your Nutritional Health (DETERMINE) checklist	2
MUST: Malnutrition Universal Screening Tool	2
MNA: Mini Nutritional Assessment	53
NRI: Nutrition risk index	2
NSI: Nutrition Screening Initiative (all)	3
SCREEN II: Seniors in the Community: Risk Evaluation for Eating and Nutrition	3
SNAQ65+: Short Nutritional Assessment Questionnaire	2
Qualitative	1
Mixed Nutritional Risk measures (reviews)	4
Other Nutritional Risk measure	4
**Diets**	470
Food groups (FG)	228
FG: Carb-heavy, starchy foods	22
FG: Cereals	32
FG: Dairy, eggs	57
FG: Fats - healthy	1
FG: Fish (not specifically PUFA-rich)	79
FG: Fruits and veg	144
FG: High fat foods	5
FG: Legumes, pulses	36
FG: Low fat, calorie	2
FG: Meat, red meat	54
FG: Nuts, seeds	22
FG: Oils and fats (all)	8
FG: Olive oil, unprocessed foods	11
FG: Processed, fast food	48
FG: PUFA: polyunsaturated fatty acid rich foods	17
FG: Snacks and sweets	54
FG: Sweetened beverages	44
FG: Mixed Food Group measures (reviews)	2
FG: Other Food Group measure	6
Dietary patterns	313

DP: Adherence to diet recommendations	36
DP: Animal Source Foods (low or high)	14
DP: Breakfast	30
DP: Carb (low or high)	4
DP: Fruit/veg (low or high)	17
DP: Glycemia (load/index), insulin	11
DP: Healthy	75
DP: High protein (paleo, ketogenic)	4
DP: Low-fat, low calorie	6
DP: Meal frequency, skips, fasting	10
DP: Nutrient adequacy	2
DP: Processed, sweets, fast food	26
DP: Traditional	26
DP: Unhealthy	28
DP: Varied, diverse	3
DP: Vegetarian, vegan, omni	24
DP: Western, modern	26
DP: DASH: Dietary Approaches to Stop Hypertension	7
DP: DII: Dietary Inflammatory Index	19
DP: Mediterranean diet	47
DP: Dietary diversity (Minimum Dietary Diversity, Individual Dietary Diversity Score)	4
DP: Dietary Variety Score	2
DP: DHQ (all): Diet History Questionnaire	2
DP: DQI (all): Diet Quality Index	7
DP: DQS: Diet Quality Score	4
DP: GDI: Global Dietary Index	1
DP: HEI (all): Healthy Eating Index	7
DP: Vegetable Variety Score	1
DP: Factor analysis (Exploratory Factor Analysis, Performance Factor Analysis)	56
DP: Mixed Dietary Pattern measures (reviews)	19
DP: Other Dietary Pattern measure	13
Diets: qualitative	9
**Nutrient, supplement intakes**	277
Vitamins (V)	110
V: Retinoids, Carotene (Vit A)	25
V: B1 - Thiamine	25
V: B2 - Riboflavin	27
V: B3 - Niacin	20
V: B5 - Pantothenic acid	7
V: B6 - Pyridoxine	35
V: B12 - Cobalamin	35
V: Biotin	4
V: C - ascorbic acid	32
V: D - calciferol	44
V: E -Alpha-tocopherol	13
V: B9 - Folic acid, folate	56
V: K - Phylloquinone, menadione	2
Minerals (M)	81
M: Calcium	33
M: Chromium	1

M: Copper	8
M: Fluoride	2
M: Iodine	4
M: Iron	34
M: Magnesium	30
M: Manganese	6
M: Phosphorus	8
M: Potassium	10
M: Selenium	9
M: Sodium	14
M: Zinc	42
Macronutrients (Ma)	152
Ma: Total caloric intake	51
Ma: Carbohydrates	26
Ma: Sugars	8
Ma: Protein	33
Ma: Fibre	27
Ma: Fats, cholesterol	51
Ma: Fatty acids (Healthy)	94
Polyphenols, antioxidants, flavonoids	11
Supplements	87
Mixed Nutrient Intake measures (reviews)	9
Other Nutrient Intake measure	2
**Nutrient Biomarkers**	202
Vitamins (V)	112
V: Retinoids, Carotene (Vit A)	4
V: B1 - Thiamine	1
V: B2 - Riboflavin	3
V: B6 - Pyridoxine	6
V: B12 - Cobalamin	22
V: C - ascorbic acid	4
V: Choline	1
V: D - calciferol	74
V: E -Alpha-tocopherol	3
V: B9 - Folic acid, folate	28
Minerals (M)	35
M: Calcium	3
M: Iodine	2
M: Iron	16
M: Magnesium	2
M: Phosphorus	2
M: Selenium	2
M: Zinc	11
Macronutrients (Ma)	64
Ma: Sugars	3
Ma: Protein	4
Ma: Fats, cholesterol	26
Ma: Fatty acids (Healthy)	40
Polyphenols, antioxidants, flavonoids	2
Mixed Nutrient Biomarker measures (reviews)	3

Other Nutrient Biomarker measure	3
**Infant and Young Child Feeding (IYCF)**	124
Breastfeeding	114
Complementary foods	5
Minimum Dietary Diversity (child)	3
Minimum Meal Frequency	4
Minimally Adequate Diets	2
Qualitative	2
Mixed IYCF measures (reviews)	6
**Birth Outcomes**	245
Birth Weight	207
Birth length	29
Head circumference	24
SGA: Small-for-Gestational Age/ IUGR: Intrauterine growth restriction	63
Mixed Birth Outcome measures (reviews)	20
**Anthropometry**	783
BMI	703
Body composition	59
Body ratios, WHR	26
Calf, thigh circumference	6
MUAC: Mid-Upper Arm Circumference	7
Waist, hip circumference	86
Relative height, stunting, HAZ	44
Relative weight, wasting, WHZ	29
Underweight, WAZ	35
Mixed Anthropometry measures (reviews)	8
Other Anthropometry measure	1
**MEASURES OF MENTAL HEALTH**	
**Depression**	1186
BDI/Beck: Beck Depression Inventory	84
BDSRS: Birleson Depression Self-Rating Scale	2
Burnam-8 Depression scale	3
CDI: Child Depression Inventory	24
CES-D: Centre for Epidemiological Studies- Depression scale	332
DASS - dep only	2
DSM symptom screen: Diagnostic Schedule of Mental Health screeners	3
EPDS: Edinburgh Postpartum Depression Scale	183
Euro-D: European Depression Screener	2
GDS: Geriatric Depression Scale	105
Goldberg	7
HADS-D: Depression subscale of the Hospital Anxiety and Depression Scale	7
HDRS, HAM-D: Hamilton Depression Rating Scale	15
Hopkins - depression only	4
HPL: Human Population Laboratory Depression Scale	5
IDS-SR30: Inventory of Depressive Symptomatology, Self-Report 30 questions	10
Kandel, Davies Scale	4
Kessler depression only: Kessler Psychological Distress Scale	1

MADRS: Montgomery-Asberg Depression Rating Scale	6
PHQ-9, PHQ: Patient Health Questionnaire	104
Zung scale	23
Single question screens	28
Self report of diagnosis	85
GWAS: Genome-Wide Association Studies	8
Rx: Prescriptions as proxy for diagnosis	37
Medical records	40
Clinical interview (SCID-I)	95
Mixed Depression measures (reviews)	89
Other Depression measure	35
**Hybrid/multiple domains**	514
BASC, BESS: Behaviour Assessment System for Children, Behavioural and Emotional Screening System	2
BABS: Bradburn's Affect Balance Scale	2
Brief Symptom Inventory	5
CCHS module: Canadian Community Health Survey mental health module	2
Child Behaviour Checklist	41
HSQ - NHANES: Current health status questionnaire	3
DASS: Depression, Anxiety and Stress Scale	33
GHQ: General Health Questionnaire	76
GSHS: Global School Health Survey	3
HADS: Hospital Anxiety and Depression Scale	36
HSCL: Hopkins Symptoms Checklist	13
K-SADS-PL: Affective Disorder and Schizophrenia for School-age children, Present and Life time version	4
Malaise Inventory, Rutters	13
MH index - 5-item: 5 question Mental Health Index	6
MH inventory - 5 item: Mental Health Inventory 5 items	3
MINI - all: Mini International Neuropsychiatric Interview	7
Moods and Feelings Questionnaire	2
OCHS, R module: Ontario Child Health Study module on mental health	4
PANAS: Positive and Negative Affect Scale	6
POMS, POMS-SF: Profile of Mood States	17
PRIME-MD: Primary Care Evaluation of Mental Disorders	4
PROMIS: Patient-Reported Outcomes Measurement Information System	2
SCL - all: Symptoms Checklist	11
SDQ: Strengths and Difficulties Questionnaire	28
SRQ-20: 20-item Self-Reporting Questionnaire	25
YASR, YSR: Youth self-report, youth and adolescents self report	9
CIDI-SF: Composite International Diagnostic Interview – Short Form	14
Single question screens	9
Self/parent report	4
Rx: Prescriptions as proxy for diagnosis	3
Clinical Interview	70
Qualitative	4
Mixed Hybrid measures (reviews)	58
Other Hybrid measure	41

**Anxiety**	204
Beck Anxiety Inventory	12
Children's Manifest Anxiety Scale	2
GAD, GAD-7: General Anxiety Disorder Scale	9
Goldberg Anxiety Scale	6
HADS-A: Hospital Anxiety and Depression Scale - Anx only	10
HAM-A: Hamilton Anxiety Scale	6
MINI : Mini-International Neuropsychiatric Interview (anxiety only)	2
Prenatal Anxiety	8
SCARED: Screen Scale for Child Anxiety Related Emotional Disorders	3
STAI: State-Trait Anxiety Inventory	64
Zung, SAS: Self-reported anxiety scale	9
Single question screen	7
Self report of diagnosis	31
Rx: Prescriptions as proxy for diagnosis	3
Medical records	11
Clinical interview	15
Mixed Anxiety measures (reviews)	14
Other Anxiety measure	11
**Stress**	232
ASQ: Adolescent Stress Questionnaire	2
CRISYS: Crisis in Family Systems	3
DHS: Daily Hassles Scale	2
EMA: Ecological Momentary Assessment	1
Holmes, Rahe stress scale	2
IDPESQ-14: Indice de détresse psychologique –Enquête Santé Québec	1
Kessler stress inventory	46
Life event inventories	21
PDQ: Prenatal Distress Questionnaire	3
PHQ - Stress: Patient Health Questionnaire, stress only	2
Pregnancy, prenatal stress	7
PSI, PSI-SF: Parenting Stress Index, Short Form	4
PSS: Perceived Stress Scale	84
PSQ: Perceived Stress Questionnaire	2
Tilburg PDS: Pregnancy Distress Scale	1
Single question screen	23
Multiple scales, items combined	6
Clinical diagnosis, treatment	1
Qualitative	4
Mixed Stress measures (reviews)	8
Other Stress measure	27
**Mental wellbeing**	229
AQoL-6D: Assessment of Quality of Life 6 dimensions	3
Cantril Ladder	2
CASP 19: Control, Autonomy, Self-realisation and Pleasure scale	4
CHQ (PF, SR): Child Health Questionnaire - Parent Form, self-report, etc.	3
EQ-5D: EuroQol 5 dimension questionnaire	17
HRQoL: Health-Related Quality of Life Scale	6
IWQoL, Lite, Kids: Impact of Weight on Quality of Life	1

KIDSCREEN (all): Screening for and Promotion of Health-Related Quality of Life in Children and Adolescents	13
Kindl - all	2
Life Evaluation Index	4
Menopause Quality of Life	1
Nottingham Health profile	2
Positive and negative (+/-) experiences scale	4
Peds QoL Inventory: Paediatric Quality of Life Index	12
SF-36, Rand-36: Short Form 36 Health Survey Questionnaire	83
SF-12: Short Form 12 Health Survey Questionnaire	16
SWLS: Satisfaction with Life Scale	3
WEMWBS: Warwick-Edinburgh Mental Wellbeing Scale	3
WHO-5: World Health Organisation Five Well-Being Index	8
WHOQoL: World Health Organization Quality of Life Scale	11
WHQ: Women's Health Questionnaire	2
Single question screens	7
Qualitative	6
Mixed Wellbeing measures (reviews)	3
Other Wellbeing measure	33

**Analysis**	
Not adjusted	364
Adjusted	1420
Review: Synthesis/Pooled	83
Review: Descriptive (no synthesis)	78
**Hypothesis direction**	
Food security, nutrition → mental health	1291
Mental health → Food security, nutrition	600
Bi-directional	54
**Study design**	
Systematic Review	142
Meta analysis	69
Experimental	65
Quasi-experimental	20
Longitudinal	717
Cross-sectional	893
Case-control	72
Mixed methods	4
Qualitative	10
Ecological	1
Nested design	169
**Sample size**	
11-100	118
101-500	443
501-1000	248
1001-5000	545
>5000	591
Reviews: number of included studies	
R: 1-5	5
R: 6-10	32
R: 11-15	34
R: 16-20	19
R: 21-30	30
R: 31-40	14
R: 41-50	10
R: 51-100	15
R: >100	4
**Population: Food security and nutrition**	
Children under 5 (U5)	423
U5: Newborns	343
U5: 6-24 months	113
U5: 24-60 months	83
Children 5-12	201
Adolescents (13-18)	228
Pregnant women, mothers (PWM)	163
PWM: Pregnant	54
PWM: Postnatal	15
PWM: Perinatal	49
PWM: Mothers	15
PWM: Other (prenatal, mixed, etc)	34
Parents	7
Household	107
Male	42
Female	158
Adults (General and representative)	735
Mid to later life populations only	408
Lifetime/ life course	47
**Population: mental health**	
Children under 5	106
Children 5-12	181
Adolescents (13-18)	234
Pregnant women, mothers (PWM)	503
PWM: Pregnant	170
PWM: Postnatal	98
PWM: Perinatal	86
PWM: Mothers	80
PWM: Other (prenatal, mixed, etc)	67
Parents	27
Male	42
Female	165
Adults (General and representative)	784
Mid to later life populations only	416
Lifetime/ life course	48
**Region**	
Global	160
Low and Middle-Income Countries (LMIC)	446
Organisation for Economic Co-operation and Development countries (OECD)	1329
Africa	81
Asia	418
Arab Countries	24
Europe (all)	521
European Union	336
Non-EU Europe	192
Oceania	160
North America	549
Central America	18
South America	67
**Country**	
Algeria	1
Argentina	1
Australia	136
Austria	8
Bahrain	1
Bangladesh	12
Barbados	1
Belgium	5
Botswana	2
Brazil	52

Bulgaria	2
Burkina Faso	1
Canada	75
Chile	3
China	77
Colombia	4
Congo	1
Cote d'Ivoire	2
Croatia	2
Cuba	1
Cyprus	2
Czech Republic	1
Denmark	14
Djibouti	1
Dominican Republic	1
Ecuador	2
Egypt	5
Estonia	1
Ethiopia	18
Fiji	1
Finland	38
France	26
Germany	46
Ghana	19
Greece	14
Grenada	1
Guatemala	2
Guyana	1
Hong Kong	6
Hungary	1
Iceland	5
India	29
Indonesia	5
Iran	75
Iraq	1
Ireland	14
Israel	5
Italy	31
Jamaica	2
Japan	77
Jordan	4
Kenya	4
Korea - South (Republic of Korea)	53
Kuwait	1
Lebanon	7
Libya	1
Lithuania	3
Luxembourg	1
Malawi	4
Malaysia	12
Maldives	1
Mauritania	1
Mexico	15
Morocco	2
Myanmar (Burma)	1
Nepal	6
Netherlands, The	62
New Zealand	23
Nicaragua	1
Nigeria	3
Norway	36
Oman	2
Pakistan	11
Palestine	2
Peru	5
Poland	10
Portugal	9
Puerto Rico	1
Qatar	1
Russia	4
Rwanda	2
Saudi Arabia	3
Scotland	5
Serbia	2
Seychelles	1
Singapore	10
Slovenia	1
Somalia	1
South Africa	21
South Sudan	1
Spain	46
Sri Lanka	2
St Lucia	1
St. Vincent and Grenadines	1
Suriname	1
Sweden	29
Switzerland	11
Syria	1
Taiwan	21
Tanzania	4
Thailand	3
Tunisia	3
Turkey	18
Uganda	4
United Arab Emirates (UAE)	6
United Kingdom	113
United States of America	464
Venezuela	2
Vietnam	8
West Indies	1
Yemen	1
Zambia	3

Publication year

2000	19
2001	10

2002	17
2003	23
2004	24
2005	29
2006	40
2007	46
2008	53
2009	70
2010	90
2011	101
2012	119
2013	129
2014	148
2015	164
2016	147
2017	179
2018	212
2019	210
2020	115

Supplementary results 2: Number and proportion of studies included in the EGM by design

**Study design**	**Number**	**Percent (of 1945)**
Systematic Review	142	7%
Meta-analysis	69	3%
Reviews with meta-analysis	48	2%
Experimental	65	3%
Quasi-experimental	20	1%
Longitudinal	717	37%
Cross-sectional	893	46%
Case-control	72	4%
Mixed methods	4	<1%
Qualitative	10	<1%
Ecological	1	<1%
Nested design	169	9%

Supplementary results 3

Supplementary results 3A: Heat map of percentage of studies only from HIC countries (n=1499), colour density scaled proportionally

	Food scarcity	Diets	Nutrient intakes	Nutrient Biomarkers	Infant and Young Child Feeding	Birth Outcomes	Anthropometry
Depression	5%	15%	12%	9%	5%	6%	23%
Hybrid domains (depression+)	3%	5%	3%	2%	1%	4%	11%
Anxiety	1%	2%	1%	0%	2%	3%	3%
Stress	2%	3%	1%	1%	1%	2%	5%
Mental wellbeing	1%	4%	1%	1%	0%	1%	6%

Supplementary results 3B: Heat map of percentage of studies only from LMIC countries (n=446), colour density scaled proportionally

	Food scarcity	Diets	Nutrient intakes	Nutrient Biomarkers	Infant and Young Child Feeding	Birth Outcomes	Anthropometry
Depression	11%	11%	6%	9%	6%	7%	24%
Hybrid domains (depression+)	5%	9%	2%	2%	1%	4%	11%
Anxiety	0%	2%	1%	1%	1%	2%	3%
Stress	2%	2%	1%	1%	0%	2%	4%
Mental wellbeing	2%	1%	0%	0%	0%	0%	3%

Supplementary results 4: Number of studies published since 2000, by design

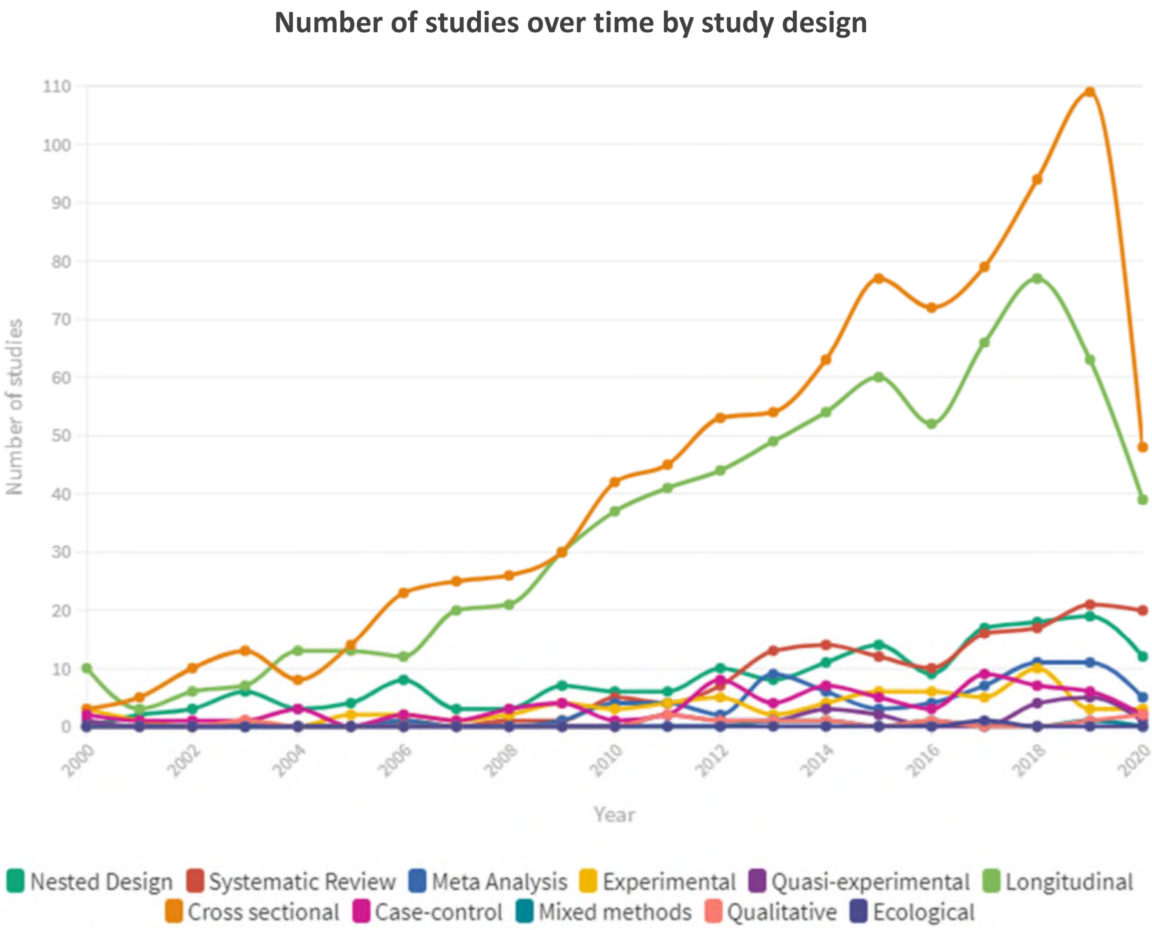

Supplementary discussion 1: Summary of opportunities for future directions in research on mental health to food security and nutrition

Box 1: Key opportunities for future research linking mental health to food security and nutrition

- Mental health dimensions: Research that focuses on anxiety, stress, and mental wellbeing, beyond depression alone
- FSN dimensions: Research on nutrients other than Vitamin D (e.g., selenium, antioxidants), as well as infant and young child feeding, child growth and cognitive development, especially research on these measures as exposures to long-term outcomes
- Study design: Studies using meta-analysis, experimental and quasi-experimental studies on FSN measures other than nutrients; mixed-methods and qualitative research
- Geography: Research on mental health and FSN relationships in low-income countries, least-developed settings, fragile and conflict-affected communities
- Populations: Research is especially needed that examines FSN as exposures to mental health outcomes for caregivers and adults; research on fathers and parents. Research is also lacking for women’s health beyond their reproductive roles.
- Shared and underlying determinants common to both mental health and FSN, especially inequity, empowerment, social support and cohesion, and instability or conflict.
