# Supplementary material for: Systematic evidence and gap map of research linking food security and nutrition to mental health
Source: Nat Commun. 2022 Aug 8;13:4608. doi: 10.1038/s41467-022-32116-3 (PMC9359994; doi:10.1038/s41467-022-32116-3)
Supplement: Supplementary file 3 — Reporting Summary [file 41467_2022_32116_MOESM3_ESM.pdf]

nature portfolio

Corresponding author(s): Thalia M Sparling
Last updated by author(s): 27/05/22

Reporting Summary

Nature Portfolio wishes to improve the reproducibility of the work that we publish. This form provides structure for consistency and transparency in reporting. For further information on Nature Portfolio policies, see our Editorial Policies and the Editorial Policy Checklist.

Statistics

For all statistical analyses, confirm that the following items are present in the figure legend, table legend, main text, or Methods section.

n/a	Confirmed	
☐	☒	The exact sample size (n) for each experimental group/condition, given as a discrete number and unit of measurement
☒	☐	A statement on whether measurements were taken from distinct samples or whether the same sample was measured repeatedly
☒	☐	The statistical test(s) used AND whether they are one- or two-sided<br>*Only common tests should be described solely by name; describe more complex techniques in the Methods section.*
☒	☐	A description of all covariates tested
☒	☐	A description of any assumptions or corrections, such as tests of normality and adjustment for multiple comparisons
☒	☐	A full description of the statistical parameters including central tendency (e.g. means) or other basic estimates (e.g. regression coefficient) AND variation (e.g. standard deviation) or associated estimates of uncertainty (e.g. confidence intervals)
☒	☐	For null hypothesis testing, the test statistic (e.g. F, t, r) with confidence intervals, effect sizes, degrees of freedom and P value noted<br>*Give P values as exact values whenever suitable.*
☒	☐	For Bayesian analysis, information on the choice of priors and Markov chain Monte Carlo settings
☒	☐	For hierarchical and complex designs, identification of the appropriate level for tests and full reporting of outcomes
☒	☐	Estimates of effect sizes (e.g. Cohen's d, Pearson's r), indicating how they were calculated

Our web collection on statistics for biologists contains articles on many of the points above.

Software and code

Policy information about availability of computer code

Data collection	The study was conducted using the EPPI Reviewer Web Beta version and EPPI Reviewer 4 platform, which was the repository for all search results, screening and coding of included studies.
Data analysis	Data was analysed with Microsoft Excel 16 and EPPI Reviewer 4 and the Web Beta version. The graphics were created in the web-based Flourish Studio platform.

For manuscripts utilizing custom algorithms or software that are central to the research but not yet described in published literature, software must be made available to editors and reviewers. We strongly encourage code deposition in a community repository (e.g. GitHub). See the Nature Portfolio guidelines for submitting code & software for further information.

Data

Policy information about availability of data

All manuscripts must include a data availability statement. This statement should provide the following information, where applicable:

- Accession codes, unique identifiers, or web links for publicly available datasets
- A description of any restrictions on data availability
- For clinical datasets or third party data, please ensure that the statement adheres to our policy

All scientific reports included in the Evidence and Gap Map were identified via Web of Science, PsychInfo, and CAB Abstracts Global Health repositories. The dataset (essentially included studies) generated during the current study are available within the HTML Evidence and Gap Map, and analysed within the manuscript and supplementary files. The full database (including initial search results and screening codes) can be accessed upon reasonable request from the corresponding author, as this is contained within EPPI Reviewer software which requires a user account.

Field-specific reporting

Please select the one below that is the best fit for your research. If you are not sure, read the appropriate sections before making your selection.

☐ Life sciences ☒ Behavioural & social sciences ☐ Ecological, evolutionary & environmental sciences

For a reference copy of the document with all sections, see nature.com/documents/nr-reporting-summary-flat.pdf

Behavioural & social sciences study design

All studies must disclose on these points even when the disclosure is negative.

Study description	Systematic Evidence and Gap Map (EGM) following PRISMA guidelines for searching literature and screening search results. Data is presented according to the state-of-the-art guidance from 3ie and EPPI Centre.
Research sample	All published reports from Medline, CAB Global Health and PsychInfo that met inclusion criteria were included in the EGM.
Sampling strategy	We included papers published in peer reviewed journals and in English that presented empirical links between measures of food security and nutrition (FSN) and mental health (MH) in human populations from anywhere in the world. We included population-based quantitative and qualitative studies of any design. We included systematic reviews based on their eligibility criteria. Measures of FSN included those in the following domains: food security, nutritional risk, diets, nutrient intakes, nutrient biomarkers, infant and young child feeding [IYCF], birth outcomes, and anthropometry. Measures of MH included those in the following domains: depression, anxiety, stress, and mental wellbeing. The rationale for this strategy was to comprehensively identify all literature on this topic, within the inclusion criteria.
Data collection	Screening: A team of screeners were trained and double-screened reports in EPPI Reviewer on title and abstract until 85% agreement rate was reached, whereafter 85% of reports were single-screened and at least 15% (sometimes more with sensitivity checking) were double screened by a senior researcher. Patterns and disagreements were discussed and additional written guidance offered. Eligible reports based on title and abstract were reviewed in full text. We undertook a similar training process, whereby once agreement rates were reached, screeners were allowed to single screen. A third of records were double screened to ensure good sensitivity. In addition to this, several iterations of backchecking and targeted searches were re-screened throughout the process.<br><br>Coding: Data was classified through a mix of a priori and iterative coding strategies in EPPI Reviewer. Fields that were decided a priori (e.g., groups of FSN and mental health measures, countries, study designs, etc.) served to identify both trends and gaps. Iterative coding included the specific measures within FSN and mental health groups. For example, although we had pre-identified a list of common and validated measures of anxiety or depression, or food security, there were many more measures that emerged beyond initial lists. These were grouped into a code if more than one study employed the measure. We used a coding form built in EPPI Reviewer to extract data on eligible reports. Only analytical comparisons and their characteristics were considered for data extraction.<br><br>We extracted information on publication year, country (or countries) and regions, study design, hypothesized direction of association between FSN and mental health (exposure-outcome relationship) and specific categories of measures and indicators, study population characteristics and sample size, and whether the analysis was adjusted or not (with at least two covariables). For the hypothesized relationship, we coded based on the authors' stated aims and methods even for cross-sectional and qualitative studies. The 'adult' population category included any age range over 18, whereas studies with populations limited to older people (usually 60 or 65+ years old) were coded with 'mid- to later-life populations only'.<br><br>Data extraction was carried out by single coding of included studies with a full review of all data extraction forms by a second researcher and targeted sensitivity checks. Given the breadth of evidence included and the aims of an evidence and gap map, quality appraisal of individual studies was not feasible or meaningful at this stage.
Timing	We search three literature repositories from January 1 2000 until July 28 2020. We excluded results before this date as there were diminishing numbers of studies on the topic prior to this date and our aim was to present the trends in the literature.
Data exclusions	We pre-defined exclusion criteria prior to screening. We did not include grey literature in our search. Studies in populations with comorbid health conditions, such as hypertension, diabetes, HIV, or surgical patients were excluded as both the nutritional and mental health correlates of these populations is likely to be unique. We also excluded studies in populations where all participants were already identified as overweight or obese, low birth weight, having mental illness. We excluded case reports (n<10), theoretical or simulation-based modelling, studies in solely clinical setting, non-systematic reviews, theses, commentaries, and abstracts.<br><br>On FSN, we excluded studies on: dietary practices and attitudes without intake measures (e.g. eating family dinners, dieting); amino acids, hormones, single, specialized or stimulant foods (e.g. arginine, seaweed, walnuts only, coffee, caffeine, alcohol); proprietary or specialized supplement or food formulas; attitudes or preferences related to infant and young child care; preterm birth (as often an outcome of non-nutritional factors); and weight change, loss or trajectories. A full list of included and excluded measures with examples and justification are included in the manuscript Supplemental methods 3A-B.<br><br>On mental health, measures that had no experiential component were excluded. Measures of cortisol were excluded as this hormone fluctuates for various reasons besides experience of stress (e.g., early in the morning, during birth, during exercise), as well as stressful event inventories or circumstances without ascertainment of perceived impact. General happiness or satisfaction

	measures were excluded as they are not direct measures of mental health, rather an indication of heightened risks or protective factors. We also excluded general health-related quality of life focusing only on physical health without mental health components separated. Lastly, we excluded studies where common mental illness could not be disentangled from other mental illness such as psychosis, bipolar disorder, substance use, eating disorders or other mental health problems.
Non-participation	Not applicable to this study as it only includes previously published studies
Randomization	Not applicable to this study as this is an Evidence and Gap Map of previously published studies identified through a systematic search.

Reporting for specific materials, systems and methods

We require information from authors about some types of materials, experimental systems and methods used in many studies. Here, indicate whether each material, system or method listed is relevant to your study. If you are not sure if a list item applies to your research, read the appropriate section before selecting a response.

Materials & experimental systems

n/a	Involved in the study	
☒	☐	Antibodies
☒	☐	Eukaryotic cell lines
☒	☐	Palaeontology and archaeology
☒	☐	Animals and other organisms
☒	☐	Human research participants
☒	☐	Clinical data
☒	☐	Dual use research of concern

Methods

n/a	Involved in the study	
☒	☐	ChIP-seq
☒	☐	Flow cytometry
☒	☐	MRI-based neuroimaging
